# Calcineurin inhibitors may be a reasonable alternative to cyclophosphamide in the induction treatment of active lupus nephritis: A systematic review and meta-analysis

**DOI:** 10.3892/etm.2014.1669

**Published:** 2014-04-07

**Authors:** MIN YANG, MIN LI, WEI HE, BIN WANG, YONG GU

**Affiliations:** 1 Department of Nephrology, The Third Affiliated Hospital of Soochow University, Changzhou, Jiangsu 213000, P.R. China; 2 Department of Gastroenterology, The Third Affiliated Hospital of Soochow University, Changzhou, Jiangsu 213000, P.R. China; 3 Division of Nephrology, Huashan Hospital and Institute of Nephrology, Fudan University, Shanghai 200040, P.R. China

**Keywords:** calcineurin inhibitors, cyclosporine, tacrolimus, lupus nephritis, meta-analysis

## Abstract

Although the accepted standard of care during the induction treatment of active lupus nephritis (LN) has been cyclophosphamide (CYC), recent trials suggest that calcineurin inhibitors (CNIs), which include cyclosporine A (CsA) and tacrolimus (TAC), may be just as, or even more, effective and less toxic than CYC. A systematic review and meta-analysis were performed to evaluate the clinical effects of CNIs on active LN compared with those of CYC. In the present study, clinical trials that compared CNIs with CYC in the induction therapy of active LN were searched in the Cochrane Library, Ovid and PubMed databases. The clinical data on renal remission and side-effects were collected and analyzed. The relative risk (RR) and 95% confidence intervals (CIs) were calculated. As a result, six controlled trials involving 265 patients were included in the meta-analysis, four of which compared TAC (treatment group) with CYC (control group), and the other two compared CsA (treatment group) with CYC (control group). CNIs were superior to CYC for higher complete remission (RR=1.56, 95% CI 1.14–2.15, Z=2.74, P=0.006) and better overall response/total remission (RR=1.23, 95% CI 1.07–1.42, Z=2.87, P=0.004) and had fewer side-effects. Among the CNIs, TAC demonstrated more favorable results than CsA. Therefore, it was concluded that CNIs may be a reasonable alternative to CYC in the induction treatment of active LN. However, large-scale, multicenter, well-designed clinical trials should be adopted to further confirm this conclusion.

## Introduction

Lupus nephritis (LN) is a common and severe manifestation of systemic lupus erythematosus (SLE). Patients with a rapidly progressive destruction of renal parenchyma often have a worse LN prognosis (1,2). In the past 20 years, much evidence has supported that patients with active LN (International Society of Nephrology/Renal Pathology Society classes III, IV or V) may be effectively treated with corticosteroids combined with immunosuppressive drugs, for example, cyclophosphamide (CYC) or mycophenolate (MMF) (3–5). Additionally, pulsed intravenous therapy with high doses of CYC followed by quarterly dosing, combined with steroids, has long been considered the ideal strategy for inducing renal remission and preventing renal flares in patients with severe LN (6). However, a significant number of patients have refractory disease or are not able to tolerate these drugs due to significant drug-related toxicity (7).

Calcineurin inhibitors (CNIs), particularly cyclosporine A (CsA) and tacrolimus (TAC), are widely used as immunosuppressive drugs. The principle action of CNIs within T lymphocytes is the inhibition of phosphatase calcineurin (8). A number of studies have demonstrated that TAC and CsA may provide equivalent potency and safety as an induction therapy in the treatment of active LN (9–14). Given the increasing popularity of CNIs in the treatment of LN, a meta-analysis was performed in the current study to compare the efficacy and safety of CNIs with those of CYC in the treatment of active LN. This was carried out by analyzing the most recently published controlled trials, including large randomized controlled trials (RCTs), prospect cohort studies, and case-control studies.

## Materials and methods

### Search strategy

A literature search was performed of the PubMed, Cochrane Library, and Ovid databases up to April 2013. The literature search was performed using the relevant keywords of ‘cyclosporine A’, ‘CsA’, ‘tacrolimus’, ‘FK506’, ‘lupus nephritis’, ‘nephritis’ and ‘glomerulonephritis’. The search was limited to articles written in English.

### Criteria for selecting articles included in this meta-analysis

Two authors extracted information from the trials independently and disagreement was resolved by consensus. In the primary stage, the titles and abstracts were scanned to exclude any studies that were clearly irrelevant. In the secondary stage, the full texts of the remaining articles were read in order to determine whether they contained information on the topic of interest. Inclusion criteria consisted of: i) the study design was an RCT, prospect cohort study or case-control study; ii) the study was of patients with biopsy-proven LN classes III, IV or V; iii) the study compared TAC or CsA with CYC in the induction therapy of LN; and iv) at least one of the following three outcomes was reported: achievement of complete renal remission (CR), partial renal remission (PR), both at least six months after therapy, and common adverse effects including infection, gastrointestinal symptoms, transient increase in serum creatinine (SCr), glucose disorders, irregular menstruation, leucopenia and liver function disorders. Exclusion criteria were: i) abstract not in English; ii) did not compare TAC or CsA with CYC in the treatment of LN; iii) did not concern the induction treatment of LN; iv) studies including children.

### Data extraction

The same reviewers independently extracted data from all primary studies that fulfilled the inclusion criteria, with disagreement resolved by consensus. Data extracted included study design, details of treatment protocol, baseline demographics and clinical, laboratory and renal biopsy information. Data on the following outcomes were extracted when reported: CR, PR, total renal remission (TR) and adverse effects.

### Outcome measures of this meta-analysis

The primary outcomes were the proportion of patients who achieved CR, PR and TR at six months or later after induction therapy with CNIs or CYC. The secondary outcomes were the relative risks (RRs) of the adverse effects including infection, gastrointestinal symptoms, transient increase in SCr, glucose disorders, irregular menstruation, leucopenia and liver dysfunction at the end of the respective studies. Definitions of the primary outcomes used in the original papers were extracted as described in Table I.

### Assessment of trial quality

The quality of each RCT was assessed using a standard scoring system proposed in the Jadad scale criteria (15). These included: i) whether the randomization method was appropriate; ii) whether double-blindness was mentioned in the trial and whether it was appropriately performed; iii) whether the description (the patient number and reasons) of withdrawal and drop-outs was clearly stated. The studies were classified as high quality if they scored >2. Otherwise, they were classified as low quality (16,17). The quality of the cohort and case-control studies was assessed using the Newcastle-Ottawa Scale (NOS) with certain modifications to match the requirements of the current study (18). The quality of the studies was evaluated by examining three items: patient selection, comparability of CNI and CYC groups, and assessment of outcomes. For the comparability between the CNI and CYC groups, the focus was on the following variables: age, gender, proteinuria, serum albumin, SCr, estimated glomerular filtration rate (eGFR) or creatinine clearance rate, serum complement component 3, anti double-stranded DNA antibodies, systemic lupus erythematosus disease activity index (SLEDAI), pathological type, pathological activity index and pathological chronicity index. Studies were graded on an ordinal star scoring scale with higher scores representing studies of a higher quality. The quality of each study was graded as either level one (0–5 stars) or level two (6–9 stars).

### Statistical analyses

All statistical analysis was performed using Stata software, version 11.0 (StataCorp LP, College Station, TX, USA). The fixed-effects model of Mantel-Haenszel was used to estimate the pooled RR with 95% confidence intervals (CIs) for study outcomes, using data from all eligible papers. The possibility of heterogeneity in results across the studies was examined using the H statistic and I2 index (19). Heterogeneity was considered statistically significant if P<0.1 in the χ^2^ test. If no statistical heterogeneity existed among the trials, a fixed-effects model was selected to analyze the data. When statistical heterogeneity was detected, the sources of heterogeneity were explored and subgroup analyses were performed. The robustness of the pooled effect sizes was evaluated based on the different types of CNIs (i.e., TAC vs. CsA) or study types (i.e., RCT vs. Non-RCT). P<0.05 was considered to indicate a statistically significant difference.

## Results

### Study selection

**Figure 1:**
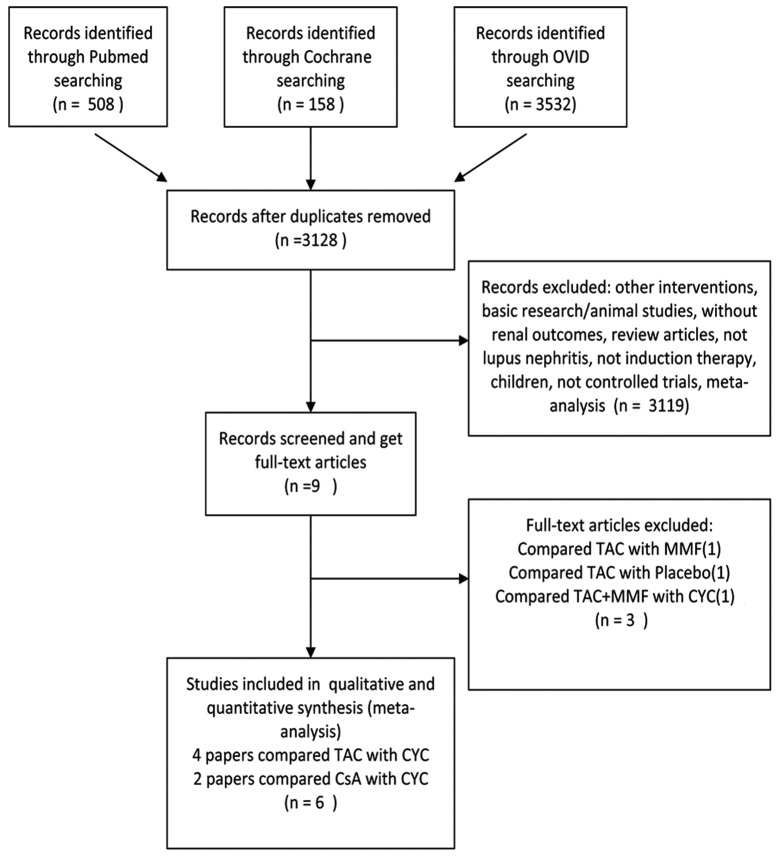
Flow diagram of studies considered for inclusion. TAC, tacrolimus; MMF, mycophenolate; CYC, cyclophosphamide; CsA, cyclosporine A.

Initially, 5,815 articles were identified through database searches. All abstracts were scanned and nine studies (20–22) were retrieved for detailed scrutiny. Thus, 5,806 abstracts were rejected on initial screening (Fig. 1). Three publications were further excluded as one compared TAC with a placebo (20), one compared TAC with mycophenolate mofetil (MMF) (21) and one compared TAC plus MMF with CYC (22). The definitive analyses in the present meta-analysis included four RCTs (9,11,13,14), one cohort study (10) and one case-control study (12) that were published between 2008 and 2013.

### Trial characteristics and qualities

Table II shows the characteristics of the papers that were included in the meta-analysis. A total of 265 patients had been assessed in the six studies. Of the six studies, four provided data for comparing the efficacy of TAC plus a steroid with that of CYC plus a steroid (9–11), and two compared the efficacy of CsA plus a steroid with that of CYC plus a steroid in the induction therapy of active LN (13,14). For the quality of the RCTs, as assessed by the Jadad method (15), the Jadad score ranged from 2 to 4 and only one trial was of high quality (Jadad score = 4). For the quality of the cohort and case-control studies, as assessed by the NOS (18), the NOS score ranged from 5 to 8 stars.

### Trial outcomes

#### Comparison of the CNI regimen with the CYC regimen

**Figure 2:**
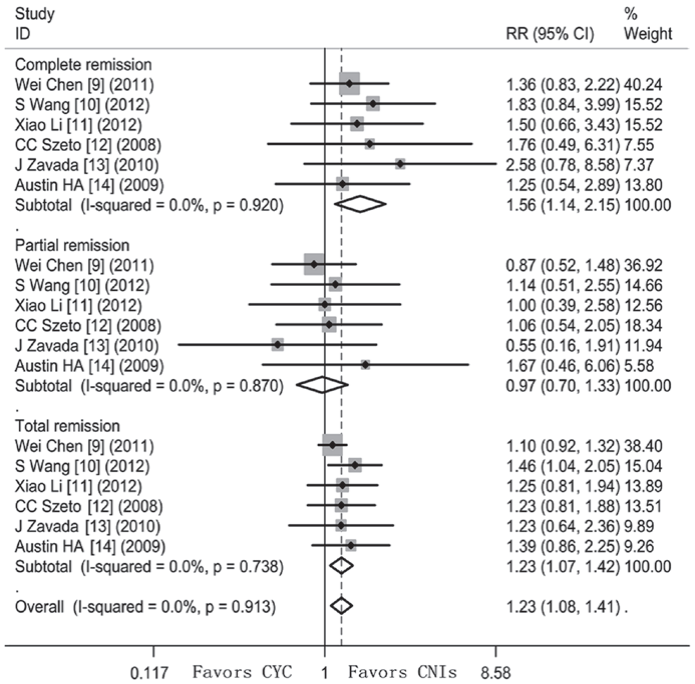
Forest plot of the relative risks for complete remission, partial remission and total remission for calcineurin inhibitors (CNIs) versus cyclophosphamide (CYC) in the induction treatment of lupus nephritis at the end of the original study duration. RR, relative risk; CI, confidence interval.

CNIs increased the rates of the following compared with CYC (Fig. 2): CR (RR=1.56, 95% CI 1.14–2.15, Z=2.74, P=0.006; heterogeneity χ^2^=1.44, P=0.920, I^2^=0%) and TR (RR=1.23, 95% CI 1.07–1.42, Z=2.87, P=0.004; heterogeneity χ^2^=2.76, P=0.783, I^2^=0%). However, PR did not reach a significant difference between CYC and CNIs (RR=0.97, 95% CI 0.70–1.33, Z=0.20, P=0.844; heterogeneity χ^2^=1.85, P=0.870, I^2^=0%).

#### Adverse events

**Figure 3:**
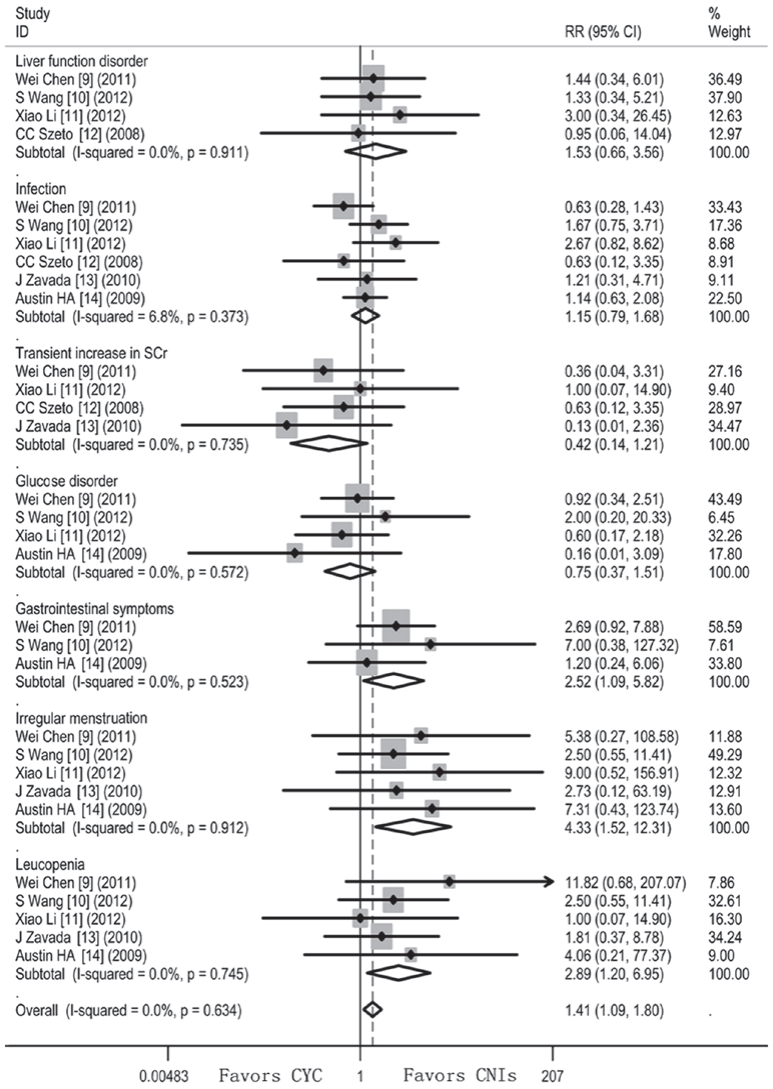
Forest plot of the relative risks for adverse events for calcineurin inhibitors (CNIs) versus cyclophosphamide (CYC) in induction treatment of lupus nephritis at the end the of original study duration. RR, relative risk; CI, confidence interval.

Significantly fewer patients who received CNIs developed irregular menstruation (RR=4.33; 95% CI 1.52–12.31), gastrointestinal disorder (RR=2.52; 95%CI 1.09–5.82) and leucopenia (RR=2.89; 95% CI 1.20–6.95). However, patients receiving CNIs appeared to have a higher risk of experiencing a transient increase in SCr (RR=0.42; 95% CI 0.14–1.21) and glucose disorder (RR=0.75; 95%CI 0.37–1.51) though this did not reach statistical significance. Heterogeneity was undetectable when the effect sizes of side-effects were evaluated (I^2^=0). The fixed-effects model was thus used (Fig. 3).

#### Sensitivity analyses

The results of the primary and secondary outcomes, including the RRs of CR, PR, TR and side-effects, remained generally consistent upon sensitivity analysis based on the type of CNI (Table III). When the trials that compared CsA with CYC in the induction therapy of LN were excluded, TAC showed a better response, higher rate of inducing remission and lower risk of adverse events than CYC. This was consistent with the results of the analysis containing all the trials, although the RR values were slightly lower. CsA did not reveal a superiority when compared with CYC in the induction therapy of active LN. When only RCT trials were pooled for analysis, the incidence of gastrointestinal symptoms became insignificant. Nevertheless, these results must be interpreted with caution since the effect sizes were generated from a small number of RCTs (n=4) and trials that involved TAC (n=4) (9–12) and CsA (n=2) (13,14) (Table III).

## Discussion

LN is a major cause of mortality in patients with systemic lupus erythematosus (SLE). Despite the relatively high renal remission rate following treatment with CYC, as many as 15% of patients with LN are refractory to treatment and up to 50% of patients develop end-stage renal disease (ESRD) during the treatment (23–26). In addition, CYC is associated with significant toxicity, particularly infections, malignancy and infertility (27). Thus, novel therapeutic strategies are necessary for the improved clinical management of patients with LN. CsA and TAC have been found to be safer than, or at least as efficacious as, CYC in inducing renal remission in a number of published controlled trials (9–14).

The current meta-analysis of six trials involving 265 patients with active LN revealed that, in terms of inducing renal remission, LN patients demonstrated a better treatment response to CNIs compared with CYC. In addition, the risks of developing irregular menstruation, gastrointestinal disorder and leucopenia were significantly lower in patients undergoing induction therapy with CNIs compared with those receiving CYC. However, CNIs were associated with a higher incidence of experiencing a transient increase in SCr and glucose disorder than CYC. In the analysis of the two different CNIs, TAC was indicated to be superior, revealing a better response rate and fewer adverse side-effects than CYC. Partially due to the small sizes of the trials, CsA did not demonstrate any superiority when compared with CYC. Previously reviewed *in vivo* and *in vitro* studies have revealed that TAC is more potent than CsA in its action (28). Therefore, the results of the present study, that TAC was superior to CsA in the induction therapy of LN, were anticipated.

Any systematic review must assess the suitability of identified trials for pooling in a meta-analysis. In the present study, all trials that were included in the analyses were comparable in several respects: duration of follow-up, pathological activity and chronicity indices, and the age, gender and renal function of the patients.

The meta-analysis in the current study generally agrees with previously published RCTs and systematic reviews (29,30). Although the conclusions are based on a small number of randomized trials, they are strengthened by the homogeneity of the study results and lack of any clear publication bias. The results favoring CNIs (CR, TR and the adverse effects) strengthen the evidence for the efficacy and safety of CNIs as alternative induction agents.

Meta-analyses are applied with increasing frequency compared with RCTs; the latter are considered to provide the strongest evidence regarding an intervention (31). The use of observational data for meta-analysis is often dismissed as being inferior in quality to data from RCTs. However, in many situations, RCTs are not feasible and only data from observational studies are available. In addition, even though RCTs mainly produce convincing evidence, flaws in the design, such as performance bias and detection bias (32), may compromise the quality of the evidence that they produce. Thus, at certain times observational studies may produce more reliable evidence than RCTs. To our knowledge, >1,000 papers relating to the meta-analysis of observational studies currently exist. Furthermore, in order to eliminate the bias from observational studies, a sensitivity analysis containing RCTs only was performed in the present study and the results of the meta-analysis did not change significantly.

Despite the added precautions, there are limitations to the current study. Firstly, although all the trials included were similar in terms of the baseline characteristics of the patients, duration of follow-up, pathological activity and chronicity indices and renal function, there were also several differences, including the definitions of complete and partial remission, the dosages of CNIs and CYC used, and the route of CYC administration. These differences may have introduced bias into the analyses. This highlights the need for adequately powered RCTs and observational studies. Secondly, although statistically significant results with regards to CR, TR and several side-effects were obtained, all results were based on a relatively small number of events and may therefore have been susceptible to random error. Small meta-analyses should be regarded with caution even in the presence of statistically significant results (33). However, meta-analyses are able to combine data from trials with small sample sizes in order to obtain useful information. Thirdly, the poor quality of the trials, mainly resulting from the lack of double-blindness and double-dummy procedures, may have further compromised the validity of the results. Fourthly, the trials also differed in the ethnicity of the participants, with four studies including only Asian patients (10–13). It is known that there are significant differences in the response of different ethnic groups to treatment of LN. Previous reports have implied that African-Americans have a three-fold higher incidence rate of SLE compared with Caucasians and often develop nephritis (34,35). Hispanics and Asians also have a greater frequency and severity of nephritis compared with Caucasians (34). However, there are inadequate data in the current study to examine the treatment allocation to ethnicity interactions directly and to make any final conclusions on this topic. Finally, both proliferative (III and IV) and membranous (V) lupus nephritis were included in the current study, although the latter is felt to exhibit a relatively benign course and a better prognosis. This may have biased the final conclusions that CNIs are more effective than CYC in all patients with active LN. In the present meta-analysis, the majority of studies included LN in classes III, IV or V (9–11), whereas a smaller number of trials included only LN in class V (12,14). Among these studies, only Chen *et al* (9) performed a subgroup analysis that was restricted to patients with proliferative lesions (severe class III and class IV). The study drew the conclusion that the CR rates were similar between the TAC and CYC groups after induction treatment in patients with LN classes III or IV. However, the CR rates were higher in the TAC group than in the CYC group in patients with LN classes V, V+IV, or V+III, although this difference was not statistically significant. Owing to the lack of individual patient data in trials that included small numbers of class V LN patients, it was not possible to perform a subgroup analysis. This may have caused bias in the meta-analysis of the present study. Due to the different pathogenesis between proliferative (class III and IV) LN and membranous (class V) LN, the response to CINs may vary. The review performed by Moroni *et al* (36) revealed an important antiproteinuric effect of CsA with a cumulative rate of CR or PR approaching 90% both in patients with proliferative LN and in patients with membranous nephropathy. Similarly, Favre *et al* (37) concluded that CsA had a notable effect on proteinuria accompanying proliferative and membranous glomerulonephritis. With regards to TAC, several studies (9,12,38) reported the efficacy and absence of serious adverse effects when TAC was administered as an induction and maintenance therapy for patients with proliferative and membranous LN. Therefore, it may be hypothesized that CNIs are likely to be effective in the treatment of patients with proliferative and membranous LN. However, the question of whether CNIs were more efficacious and safer than CYC respectively for patients with proliferative and membranous LN remains uncertain. Therefore, the results of the current meta-analysis, despite being comprehensive, may not fully reflect the relative efficacy and safety profile of CNIs and CYC and the results should be interpreted with caution. Nevertheless, every effort has been implemented to eliminate potential bias and the sensitivity analyses have improved the robustness of the pooled estimates.

In conclusion, CNIs, especially TAC, may be reasonable alternative treatments for patients with active LN who are insensitive or intolerant to CYC in induction therapy.

## Figures and Tables

**Table I:** Definitions of complete remission, partial remission and total remission.

Study (ref.)	Complete remission	Partial remission	Total remission
Chen *et al* (9)	Proteinuria <0.3 g/24 h with normal urine sediment, Alb≥35 g/l, normal SCr range or not >15% more than baseline	Proteinuria range of 0.3–2.9 g/24 h and decrease ≥50% of baseline, Alb≥30 g/l, normal SCr range or not >15% more than baseline	CR or PR
Wang *et al* (10)	Proteinuria <0.5 g/24 h with normal urine sediment, Alb≥35 g/l, stable or improved eGFR≥10% for baseline SCr≥133 umol/l	Stable or improved eGFR; reduction of proteinuria≥50% of the basal level but still >0.5 g/24 h; Alb≥30 g/l (≥2 determinations one month apart)	CR or PR
Li *et al* (11)	Proteinuria <0.3 g/24 h with normal urine sediment, Alb≥35 g/l and stabilization (±15%) or improvement in SCr at 24 weeks.	Proteinuria (0.3–2.9 g/24 h) and decrease ≥50% of baseline; Alb≥30 g/l; stabilization (±30%) in SCr.	CR or PR
Szeto *et al* (12)	Proteinuria <0.5 g/24 h with normal urine sediment, normal Alb, eGFR≤15% above baseline	Proteinuria (0.5–2.9 g/24 h), Alb≥30 g/l, stable renal function	NR
Zavada *et al* (13)	proteinuria <0.3 g/24 h with normal urine sediment, SCr within the normal range with stable or not >15% more than baseline	SCr within the normal range with stable or not >15% more than baseline, proteinuria decrease ≥50% of baseline and proteinuria <3 g/24 h if nephritic at baseline or ≤0.5 g/24 h if baseline non-nephritic, normal urine sediment or C3 improvement ≥25%	CR or PR
Austin *et al* (14)	Proteinuria <0.3 g/24 h	Proteinuria <2.0 g/d and decrease ≥50% of baseline	NR

SCr, serum creatinine; Alb, albumin; eGFR, estimated glomerular filtration rate; NR, not reported; CR, complete remission; PR, partial remission.

**Table II:** Trial characteristics and qualities.

Study (ref.)	Country or area	Study type	Number enrolled	Age (years)	Comparison	Renal pathology type	Follow-up duration (months)	Jadad score /Newcastle-Ottawa Scale
Chen *et al* (9)	China	RCT	TAC 42	32±10.8	TAC+Pred vs. IV CYC+Pred	Class III, IV, V	6	4
			CYC 39	31.9±10.1				
Wang *et al* (10)	China	CS	TAC 20	32±7.7	TAC+Pred vs. IV CYC+Pred	Class III, IV, V	21.25±15.25	********
			CYC 20	35.7±11.4			16.83±15.85	
Li *et al* (11)	China	RCT	TAC 20	29 (17–50)	TAC+Pred vs. IV CYC+Pred	Class III, IV, V	6	2
			CYC 20	33 (17–64)				
Szeto *et al* (12)	Hong Kong	CC	TAC 18	38.2±10.4	TAC+Pred vs. PO CYC+Pred	Class V	6	*****
			CYC 19	36.5±12.2				
Zavada *et al* (13)	Czech Republic and Slovakia	RCT	CsA 19	30±9	CsA +Pred vs. IV CYC+Pred	Class III, IV	9	2
			CYC 21	28±5				
Austin *et al* (14)	USA	RCT	CsA 12	34 (13–56)	CsA +Pred vs. IV CYC+Pred	Class V	12	2
			CYC 15	41 (17–60)				

RCT, randomized controlled trial; CS, cohort study; CC, case-control study; TAC, tacrolimus; CYC, cyclophosphamide; CsA, cyclosporine A; Pred, prednisone; PO, per os; IV, intravenous; *, number of stars on the Newcastle-Ottawa scale star ranking.

**Table III:** Results of sensitivity analyses (trials exclusion).

	Combined RR (95% CI)									
	
Comparison	CR	PR	TR	Infections	Liver function disorder	Gastrointestinal symptoms	Transient SCr↑	Glucose disorder	Leucopenia	Irregular menstruation
TAC vs. CsA										
All trials	1.56 (1.14,2.15)^a^	0.97 (0.70,1.33)	1.23 (1.07,1.42)^a^	0.87 (0.60,1.26)	0.65 (0.28,1.52)	0.40 (0.17, 0.92)^a^	2.39 (0.82,6.90)	1.33 (0.66,2.67)	0.35 (0.14,0.83)^a^	0.23 (0.08,0.66)^a^
TAC vs. CYC	1.52 (1.06,2.17)^a^	0.98 (0.70,1.38)	1.22 (1.05,1.41)^a^	0.87 (0.54,1.40)	0.62 (0.25,1.51)	0.31 (0.11, 0.86)^a^	1.75 (0.54,5.71)	6.15 (0.32,117.21)	0.30 (0.10,0.93)^a^	0.25 (0.07,0.83)^a^
CsA vs. CYC	1.71 (0.86,3.43)	0.91 (0.38,2.15)	1.31 (0.87,1.96)	0.86 (0.48,1.54)	1.06 (0.07,15.64)	0.83 (0.46, 4.21)	7.70 (0.42,140.03)	0.44 (0.11,1.75)	0.44 (0.11,1.75)	0.20 (0.02,1.56)
RCT vs. non-RCT										
All trials	1.56 (1.14,2.15)^a^	0.97 (0.70,1.33)	1.23 (1.07,1.42)^a^	0.87 (0.60,1.26)	0.65 (0.28,1.52)	0.40 (0.17, 0.92)^a^	2.39 (0.82,6.90)	1.33 (0.66,2.67)	0.35 (0.14,0.83)^a^	0.23 (0.08,0.66)^a^
RCT	1.49 (1.04,2.13)^a^	0.91 (0.60,1.36)	1.19 (1.00,1.41)	0.91 (0.59,1.41)	0.61 (0.21,1.77)	0.47 (0.19, 1.13)	3.01 (0.75,12.09)	1.50 (0.71,3.16)	0.32 (0.11,0.95)^a^	0.16 (0.04,0.70)^a^
Non-RCT	1.81 (0.93,3.53)	1.09 (0.66,1.83)	1.35 (1.04,1.76)^a^	0.76 (0.37,1.55)	0.75 (0.19,2,93)	0.14 (0.01, 2.60)	1.58 (0.30,8.40)	0.50 (0.05,5.08)	0.40 (0.09,1.83)	0.40 (0.09,1.83)

RR, relative risk; CI, confidence interval; TAC, tacrolimus; RCT, randomized control trial; non-RCT, non-randomized control trial; CYC, cyclophosphamide; CsA, cyclosporine A; CR, complete remission; PR, partial remission; TR, total remission; SCr, serum creatinine.

a P<0.05 compared with CYC.

## References

[1] Cervera R, Khamashta MA, Font J, The European Working Party on Systemic Lupus Erythematosus (1993). Systemic lupus erythematosus: clinical and immunologic patterns of disease expression in a cohort of 1,000 patients. Medicine (Baltimore).

[2] Mok CC, Tang SS (2004). Incidence and predictors of renal disease in Chinese patients with systemic lupus erythematosus. Am J Med.

[3] Austin HA, Klippel JH, Balow JE (1986). Therapy of lupus nephritis. Controlled trial of prednisone and cytotoxic drugs. N Engl J Med.

[4] Carette S, Klippel JH, Decker JL (1983). Controlled studies of oral immunosuppressive drugs in lupus nephritis. A long-term follow-up. Ann Intern Med.

[5] Boumpas DT, Austin HA, Vaughn EM (1992). Controlled trial of pulse methylprednisolone versus two regimens of pulse cyclophosphamide in severe lupus nephritis. Lancet.

[6] Mok CC, Wong RW, Lai KN (2003). Treatment of severe proliferative lupus nephritis: the current state. Ann Rheum Dis.

[7] Bhat P, Radhakrishnan J (2008). B lymphocytes and lupus nephritis: new insights into pathogenesis and targeted therapies. Kidney Int.

[8] Sommerer C, Giese T, Meuer S, Zeier M (2010). New concepts to individualize calcineurin inhibitor therapy in renal allograft recipients. Saudi J Kidney Dis Transpl.

[9] Chen W, Tang X, Liu Q (2011). Short-term outcomes of induction therapy with tacrolimus versus cyclophosphamide for active lupus nephritis: A multicenter randomized clinical trial. Am J Kidney Dis.

[10] Wang S, Li X, Qu L (2012). Tacrolimus versus cyclophosphamide as treatment for diffuse proliferative or membranous lupus nephritis: a non-randomized prospective cohort study. Lupus.

[11] Li X, Ren H, Zhang Q (2012). Mycophenolate mofetil or tacrolimus compared with intravenous cyclophosphamide in the induction treatment for active lupus nephritis. Nephrol Dial Transplant.

[12] Szeto CC, Kwan BC, Lai FM (2008). Tacrolimus for the treatment of systemic lupus erythematosus with pure class V nephritis. Rheumatology (Oxford).

[13] Zavada J, Pesickova S, Rysava R (2010). Cyclosporine A or intravenous cyclophosphamide for lupus nephritis: the Cyclofa-Lune study. Lupus.

[14] Austin HA, Illei GG, Braun MJ, Balow JE (2009). Randomized, controlled trial of prednisone, cyclophosphamide, and cyclosporine in lupus membranous nephropathy. J Am Soc Nephrol.

[15] Jadad AR, Moore RA, Carroll D (1996). Assessing the quality of reports of randomized clinical trials: is blinding necessary?. Control Clin Trials.

[16] Moher D, Pham B, Jones A (1998). Does quality of reports of randomised trials affect estimates of intervention efficacy reported in meta-analyses?. Lancet.

[17] Kjaergard LL, Villumsen J, Gluud C (2001). Reported methodologic quality and discrepancies between large and small randomized trials in meta-analyses. Ann Intern Med.

[18] Oremus M, Oremus C, Hall GB, McKinnon MC (2012). Inter-rater and test-retest reliability of quality assessments by novice student raters using the Jadad and Newcastle-Ottawa Scales. BMJ Open.

[19] Higgins JP, Thompson SG (2002). Quantifying heterogeneity in a meta-analysis. Stat Med.

[20] Miyasaka N, Kawai S, Hashimoto H (2009). Efficacy and safety of tacrolimus for lupus nephritis: a placebo-controlled double-blind multicenter study. Mod Rheumatol.

[21] Yap DY, Yu X, Chen XM (2012). Pilot 24 month study to compare mycophenolate mofetil and tacrolimus in the treatment of membranous lupus nephritis with nephrotic syndrome. Nephrology (Carlton).

[22] Bao H, Liu ZH, Xie HL, Hu WX, Zhang HT, Li LS (2008). Successful treatment of class V+IV lupus nephritis with multitarget therapy. J Am Soc Nephrol.

[23] Valeri A, Radhakrishnan J, Estes D (1994). Intravenous pulse cyclophosphamide treatment of severe lupus nephritis: a prospective five-year study. Clin Nephrol.

[24] Mok CC, Ho CT, Siu YP (2001). Treatment of diffuse proliferative lupus glomerulonephritis: a comparison of two cyclophosphamide-containing regimens. Am J Kidney Dis.

[25] Sesso R, Monteiro M, Sato E, Kirsztajn G, Silva L, Ajzen H (1994). A controlled trial of pulse cyclophosphamide versus pulse methylprednisolone in severe lupus nephritis. Lupus.

[26] Belmont HM, Storch M, Buyon J, Abramson S (1995). New York University/Hospital for Joint Diseases experience with intravenous cyclophosphamide treatment: efficacy in steroid unresponsive lupus nephritis. Lupus.

[27] Dooley MA, Ginzler EM (2006). Newer therapeutic approaches for systemic lupus erythematosus: immunosuppressive agents. Rheum Dis Clin North Am.

[28] Scott LJ, McKeage K, Keam SJ, Plosker GL (2003). Tacrolimus: a further update of its use in the management of organ transplantation. Drugs.

[29] Lee YH, Lee HS, Choi SJ, Dai Ji J, Song GG (2011). Efficacy and safety of tacrolimus therapy for lupus nephritis: a systematic review of clinical trials. Lupus.

[30] Deng J, Huo D, Wu Q, Yang Z, Liao Y (2012). A meta-analysis of randomized controlled trials comparing tacrolimus with intravenous cyclophosphamide in the induction treatment for lupus nephritis. Tohoku J Exp Med.

[31] Midgette AS, Wong JB, Beshansky JR, Porath A, Fleming C, Pauker SG (1994). Cost-effectiveness of streptokinase for acute myocardial infarction: A combined meta-analysis and decision analysis of the effects of infarct location and of likelihood of infarction. Med Decis Making.

[32] Jones T, Evans D (2000). Conducting a systematic review. Aust Crit Care.

[33] Flather MD, Farkouh ME, Pogue JM, Yusuf S (1997). Strengths and limitations of meta-analysis: larger studies may be more reliable. Control Clin Trials.

[34] Alarcón GS, Friedman AW, Straaton KV (1999). Systemic lupus erythematosus in three ethnic groups: III. A comparison of characteristics early in the natural history of the LUMINA cohort LUpus in MInority populations: NAture vs Nurture. Lupus.

[35] Cooper GS, Parks CG, Treadwell EL (2002). Differences by race, sex and age in the clinical and immunologic features of recently diagnosed systemic lupus erythematosus patients in the southeastern United States. Lupus.

[36] Moroni G, Doria A, Ponticelli C (2009). Cyclosporine (CsA) in lupus nephritis: assessing the evidence. Nephrol Dial Transplant.

[37] Favre H, Miescher PA, Huang YP, Chatelanat F, Mihatsch MJ (1989). Ciclosporin in the treatment of lupus nephritis. Am J Nephrol.

[38] Chen W, Liu Q, Chen W (2012). Outcomes of maintenance therapy with tacrolimus versus azathioprine for active lupus nephritis: a multicenter randomized clinical trial. Lupus.

